# Lysate of Probiotic *Lactobacillus casei* DN-114 001 Ameliorates Colitis by Strengthening the Gut Barrier Function and Changing the Gut Microenvironment

**DOI:** 10.1371/journal.pone.0027961

**Published:** 2011-11-22

**Authors:** Zuzana Zakostelska, Miloslav Kverka, Klara Klimesova, Pavel Rossmann, Jakub Mrazek, Jan Kopecny, Michaela Hornova, Dagmar Srutkova, Tomas Hudcovic, Jakub Ridl, Helena Tlaskalova-Hogenova

**Affiliations:** 1 Institute of Microbiology, Academy of Sciences of the Czech Republic, Prague, Czech Republic; 2 Institute of Microbiology, Academy of Sciences of the Czech Republic, Novy Hradek, Czech Republic; 3 Institute of Animal Physiology and Genetics, Academy of Sciences of the Czech Republic, Prague, Czech Republic; 4 Institute of Molecular Genetics, Academy of Sciences of the Czech Republic, Prague, Czech Republic; French National Centre for Scientific Research, France

## Abstract

**Background:**

Probiotic bacteria can be used for the prevention and treatment of human inflammatory diseases including inflammatory bowel diseases (IBD). However, the nature of active components and exact mechanisms of this beneficial effects have not been fully elucidated. Our aim was to investigate if lysate of probiotic bacterium *L. casei* DN-114 001 (Lc) could decrease the severity of intestinal inflammation in a murine model of IBD.

**Methodology/Principal Findings:**

The preventive effect of oral administration of Lc significantly reduces the severity of acute dextran sulfate sodium (DSS) colitis in BALB/c but not in SCID mice. In order to analyze how this beneficial effect interferes with well-known phases of intestinal inflammation pathogenesis *in vivo* and *in vitro*, we evaluated intestinal permeability using the FITC-labeled dextran method and analysed tight junction proteins expression by immunofluorescence and PCR. We also measured CD4^+^FoxP3^+^ regulatory T cells proportion by FACS analysis, microbiota composition by pyrosequencing, and local cytokine production by ELISA. Lc leads to a significant protection against increased intestinal permeability and barrier dysfunction shown by preserved ZO-1 expression. We found that the Lc treatment increases the numbers of CD4^+^FoxP3^+^ regulatory T cells in mesenteric lymph nodes (MLN), decreases production of pro-inflammatory cytokines TNF-α and IFN-γ, and anti-inflammatory IL-10 in Peyer's patches and large intestine, and changes the gut microbiota composition. Moreover, Lc treatment prevents lipopolysaccharide-induced TNF-α expression in RAW 264.7 cell line by down-regulating the NF-κB signaling pathway.

**Conclusion/Significance:**

Our study provided evidence that even non-living probiotic bacteria can prevent the development of severe forms of intestinal inflammation by strengthening the integrity of intestinal barrier and modulation of gut microenvironment.

## Introduction

Inflammatory bowel diseases (IBD), such as Crohn's disease and ulcerative colitis, are severe chronic inflammatory illnesses of the gastrointestinal tract. Although their etiology and pathogenesis are not fully understood, it is generally accepted, that the inflammation is a result of an aberrant immune response to antigens of resident gut microbiota in genetically susceptible individuals [1]. Moreover, dysbiosis, an imbalance in the intestinal bacterial ecosystem, has been found in IBD and linked to its pathogenesis [2]. It has been suggested that this microbial imbalances and an aberrant immune response could be restored by oral administration of certain beneficial bacterial species, probiotics [3].

When administered in adequate amounts, probiotics, defined as live microorganisms, confer a health benefit to the host [4], and have been successfully used in treatment of IBD [5]. Using animal models of IBD, three main mechanisms of how these beneficial microbes protect from intestinal inflammation have been described. A single probiotic bacterium could possess more than one mechanism depending on its unique specific metabolic activities and cellular structures [6]. First, probiotics may exclude or inhibit the growth of certain pathogens [7]; second, they may improve the gut barrier function [8]; and third, they can modulate mucosal and/or systemic immune response or metabolic functions [9]. The outcome of probiotic therapy also depends on the stage of the disease and the overall health status of the patient. Despite of the generally safe profile of the probiotic therapy, the use of live microorganisms may lead to severe infections, and therefore represents considerable risk especially in severely ill patients [10]. There is increasing evidence, that similar beneficial effects could be achieved with sterile lysates or components isolated from probiotic or even commensal microbes [11].

Colitis induced by dextran sulfate sodium (DSS) is a well established and reliable model of IBD because its clinical features resemble the ulcerative colitis [12]. Acute DSS colitis starts with epithelial cell barrier dysfunction which causes the antigens from the gut lumen to enter the lamina propria and stimulate the immune response. The dysfunction of the epithelial barrier starts as early as the first day after DSS treatment by gradual decrease in the tight junction protein ZO-1 production, which in turn leads to increased gut permeability [13], [14]. In the acute phase, DSS-induced colitis is driven mainly by cells of innate immunity, because it also occurs in the absence of functional T, B and NK cells [15]. The functional adaptive immune system, however, plays an important role in the chronic phase of the inflammation and might be necessary for its preventive treatment with microbial antigens [11], [16].

The most intensively studied and used probiotic bacteria are lactobacilli [17], [18]. Oral treatment with probiotic bacterium *L. casei* DN-114 001 has been found to reduce the duration and severity of diarrhea and common infectious diseases in children [19]. Moreover, supernatant of this probiotic strain was described to exert immunological activities and strong inhibitory effect on epithelial cell adhesion of virulent *E. coli* strain [20] . These studies clearly show the beneficial potential of this bacterium, however, the clinical utility of such approach remains controversial, as neither the specific mechanisms of action nor the active component responsible for its beneficial properties has been established.

In our previous study, we have shown that the preventive treatment with live probiotic bacterium *L. casei* DN-114001 protects mice from subsequent acute DSS-induced colitis in BALB/c mice [21]. Here, we show that oral treatment with lysate of this bacterium (Lc) has a similar effect, and that this effect is associated with change in the intestinal microbiota composition, modulation of mucosal immune system, and induction of regulatory T cells in mesenteric lymph nodes (MLN). Our results show that even killed probiotic bacteria can decrease the severity of the intestinal inflammation, which represents safer and more practical therapeutic intervention than the use of live bacteria in the treatment of intestinal inflammation.

## Materials and Methods

### Preparation of bacteria

*Lactobacillus casei* DN-114 001 (Danone Institute, Palaiseau Cedex, France), *Lactobacillus plantarum* CCDM 185 (Culture Collection of Dairy Microorganisms, Milcom a.s., Prague), were grown in an anaerobic chamber in De Man, Rogosa, and Sharpe broth (Oxoid, Basingstoke, UK) at 37°C until the cultures were in the late log phase of growth. Both lactobacilli were harvested by centrifugation (4000× g, 30 min) and washed twice with sterile phosphate-buffered saline (PBS). After the treatment with the French press, lactobacilli were freeze-dried and diluted to a working concentration of 30 g/l. In order to kill all remaining viable bacteria, the lysate was heated to 60°C for 30 min and the sterility of all components was verified by both aerobic and anaerobic 48 hours cultivation before administration.

### Animals

Ethics statement: All animal experiments were approved by the Animal Care and Use Committee of the Institute of Microbiology, Academy of Sciences of the Czech Republic, approval ID: 10/2005, 94/2008 and 211/2009. Female BALB/c mice (8–12 weeks old) or severe combined immunodeficient mice BALB/cJHanHsd-SCID (SCID) were obtained from a breeding colony at the Institute of Physiology (Academy of Sciences of the Czech Republic, Prague, Czech Republic) or at the Institute of Microbiology (Academy of Sciences of the Czech Republic, Novy Hradek, Czech Republic), respectively, and reared under conventional conditions.

### Study design and DSS induced colitis

We administered 1.5 mg of Lc in 50 µl of sterile PBS, i.e. 6×10^8^ CFU of heat killed bacteria, by gavage. To reduce proteolytic activity in the gut, the Lc components were co-administered with 1 mg of soybean trypsin inhibitor (Sigma-Aldrich, St. Louis, MO, USA) dissolved in 50 µl of 0.15 M sodium bicarbonate buffer (pH 8.0). Control mice were given only sterile PBS with soybean trypsin inhibitor in bicarbonate buffer. The administration of lysates was repeated every 7 days for a total number of 4 doses (on days 0, 7, 14 and 21). Acute colitis was induced 7 days later by 3% (wt/v) DSS (molecular weight 36–50 kDa; MP Biomedicals, Irvine, CA, USA) dissolved in tap water for 7 days, and on the last day of the experiment the colitis was evaluated by using a clinical activity score, colon length, and the histological scoring system as described previously [11]. Furthermore, to analyze if the protective effect of Lc could be achieved also by parenteral administration, four subcutaneus doses of Lc or PBS (25 µg per dose) were injected in incomplete Freund's adjuvant (Difco Laboratories, Detroit, MI, USA) before colitis induction. For chronic colitis, mice received four cycles of DSS as described previously [12].

### Evaluation of intestinal barrier function

#### Intestinal permeability *in vivo*

The intestinal permeability was measured by determining the amount of FITC-dextran in blood after it was orally administered as described previously [22]. Briefly, each mouse received 440 mg/kg of body weight of FITC-dextran (molecular weight 4.4 kDa; Sigma-Aldrich) by gavage. A blood sample, obtained 5 h later, was first centrifuged (3,000 rpm at 4°C) for 30 min, and serum was collected and added to a 96-well microplate. The concentration of FITC-dextran was determined by spectrophotofluorometry (Safire^2^, Tecan Group Ltd., Männedorf, Switzerland) with an excitation wavelength of 483 nm and an emission wavelength of 525 nm using serially diluted samples of the marker as standard.

#### Immunohistology

Segments of colon and terminal ileum were frozen in liquid nitrogen immediately after removal and stored at −80°C until used. Frozen sections (6 µm) were mounted on the poly-L-lysine-coated slides. Then the slides were dried and fixed in 4% buffered paraformaldehyde (pH 7.4) for 10 min at room temperature. Fixed sections were washed in PBS and blocked with 2% donkey serum (Sigma-Aldrich) in PBS for 20 min at room temperature. The slides were incubated with the rabbit polyclonal anti-mouse ZO-1 or occludin antibodies (both from Invitrogen, Camarillo, CA, USA) overnight at 4°C. The negative controls were performed similarly using 1% bovine serum albumin (BSA) in PBS instead of primary antibody. After washing, the sections were incubated with donkey anti-rabbit antibody conjugated either with Texas Red or with DyLight 488 fluorochrome (both from Jackson ImmunoResearch Laboratories, West Grove, USA). Nuclei were counterstained using DAPI (4′,6-diamidino-2-phenylindole; Sigma-Aldrich) stain. Finally, the sections were mounted in Vectashield mounting medium for fluorescence (Vector Laboratories, Burlingame, CA, USA) and viewed with a fluorescence microscope Olympus AX-70 (Olympus, Tokyo, Japan).

#### Determination of ZO-1 mRNA expression in intestinal tissue

Intestinal mucosa from terminal ileum and colon was placed in RNAlater stabilization reagent (QIAGEN GmbH, Hilden, Germany). Total messenger RNA (mRNA) was extracted by using the RNeasy Mini isolation kit (QIAGEN GmbH) following the manufacturer's instructions. RNA integrity was determined by gel electrophoresis in 1.5% agarose gel stained with ethidium bromide. The purity of the RNA was assessed by the ratio of absorbance at 260 and 280 nm. RNA purity was within a range of 2.0–2.1. The total RNA concentration was estimated by spectrophotometric measurements at 260 nm assuming that 40 µg of RNA per mililitre equal one absorbance unit. Real time PCR was performed as described previously [23]. Briefly, RNA was converted to cDNA using Taq-Man reverse transcription reagents (Applied Biosystems, Foster City, CA, USA). Beta-actin was used as an endogenous control and its expression was similar in all tested samples. A reaction mix for real-time PCR was made with Taq-Man Universal PCR master mix, water, and assays on demand gene expression products for ZO-1 and β-actin (all Applied Biosystems, Foster City, CA, USA). The master mix (20 µl) was aliquoted to the wells on a real-time PCR plate; and each sample was analyzed in duplicate. A volume of 5 µl of cDNA was added to each well, and the PCR reaction was run on a 7300 real-time PCR System (Applied Biosystems, Foster City, CA, USA) using standard conditions. The data was analyzed with Genex software (version 4.3.8).

### Production of cytokines

#### Intestinal tissue culture and measurement of cytokines

Sections of Peyer's patches (PP), ileum, cecum, and colon were taken from every mouse. The intestines were then opened longitudinally, washed in ice cold PBS containing antibiotics and cultivated for 48 hours at 37°C and 5% CO_2_ in complete RPMI medium with 10% fetal bovine serum (Biochrom AG, Berlin, Germany) and 100,000 U/l penicillin, 100 mg/l streptomycin (Sigma-Aldrich), as described previously [11] . Commercially available ELISA sets were used to measure the levels of TNF-α, IFN-γ, TGF-β, IL-10 (Invitrogen Corp.) and IL-6 (R&D Systems Inc., Minneapolis, MN, USA) in these supernatants. All tests were performed according to the manufacturers' recommendations.

#### Determination of cytokine mRNA expression in intestinal tissue

The samples were processed as described above (see Determination of ZO-1 mRNA expression in intestinal tissue). Gene expression assays for IL-10, IL-6, TNF-α and β-actin were all purchased by Applied Biosystems, Foster City, CA, USA.

### Determination of specific antibodies

Sera and small intestine washings were collected for specific antibody evaluation. Gut washings were obtained by flushing the content of isolated small intestine with 2 ml of sterile PBS containing a mixture of proteinase inhibitors (Sigma-Aldrich). The samples were then vortexed and centrifuged at 4°C, and the supernatant was collected and stored at −80°C until analysis. Indirect ELISA, optimized in our laboratory as previously described [11], was used to assess the specific antibody response against Lc in serum (IgG, IgM, and IgA) and gut washings (secretory IgA; SIgA). Briefly, Nunc MaxiSorp 96-well plates (Thermo Fisher Scientific Inc., Rochester, NY, USA) were coated overnight with Lc (100 µl/well at 10 mg/l in PBS) and blocked with 1% BSA (Sigma-Aldrich) in PBS. Serum and gut washing samples diluted 1∶50 and 1∶10 in 1% BSA, respectively, were added and incubated for 2 hours. As control sera normal reference serum purchased from Bethyl Laboratories (TX, USA) and hyperimmune serum prepared by four subcutaneous injections of Lc in incomplete Freund's adjuvant within 14 days intervals (50 µg of Lc in the each dose) were used. After washings (three times with PBS containing 0.05% Tween 20 (Sigma-Aldrich)), secondary antibodies (50 µl/well) were added and incubated for 1 hour at room temperature. Antibody combinations were used as follows: 1) rabbit anti-mouse SIgA (Uscn Life Science Inc., China) and horseradish peroxidase (HRP)-labeled anti-rabbit IgG (Cell Signaling Technology Inc., Danvers, MA, USA); 2) biotinylated anti-mouse IgA (Sigma-Aldrich) and streptavidin-HRP (R&D Systems Inc.); 3) HRP-labeled anti-mouse IgG; 4) HRP-labeled anti-mouse IgM (both The Binding Site Ltd, Birmingham, UK). All reagents were diluted in 1% BSA in PBS except anti-IgA antibody that was diluted in 1% BSA with 5% fetal bovine serum (BioClot GmbH, Aidenbach, Germany). The plates were developed with 3,3′,5,5′-tetramethylbenzidine (Sigma-Aldrich) and the optical density (OD) was measured at 450 nm. The OD of the background (1% BSA) was subtracted and resulting adjusted ODs of the treated groups were compared with those of PBS-treated groups.

### Flow cytometry

Single-cell suspensions of spleens, MLNs and PPs were prepared and stained for T_regs_ using FoxP3 Staining Set (eBioscience, San Diego, CA, USA) with fluorochrome-labeled anti-mouse mAbs: CD4-Qdot® 605 (Invitrogen, Carlsbad, CA, USA), CD8-BD Horizon™ V500 (BD Biosciences, San Jose, CA, USA), CD3-FITC and FoxP3-Phycoerythrin (both from eBioscience) according to the manufacturer's recommendation.

RAW 264.7 cells were cultivated and stained for IL-7R-Alexa647 (a gift from Pavel Otahal, IMG AS CR, Prague, Czech Republic), CD206-PE (AbD Serotec, Oxford, UK), CD-11c-NC625 and F4/80-APC780 (both from eBioscience). Hoechst 33342 (Sigma-Aldrich) was used to determine cell viability. Flow cytometric analysis was performed on LSRII (BD Biosciences), and the data was analyzed using FlowJo software (Tree Star Inc., Ashland, OR, USA).

### Evaluation of the anti-inflammatory properties of Lc *in vitro*

The LPS-activated macrophage cell line (RAW 264.7; ATCC TIB-71) was cultivated in the presence of different concentrations of bacterial lysate, as previously described [11]. Briefly, the cells were cultured for 24 hour at 37°C and 5% CO_2_ in Dulbecco's modified Eagle's medium (Institute of Molecular Genetics AS CR, Prague, Czech Republic) containing 10% heat-inactivated fetal bovine serum (Biochrom AG), 4.5 g/l glucose, 1.5 g/l sodium bicarbonate, 4 mM glutamine (Institute of Molecular Genetics AS CR), 100,000 U/l penicillin and 100 mg/l streptomycin (both Sigma-Aldrich). The cells were cultured together with Lc, lysate of *L. plantarum* (Lp) or sterile PBS in the presence or absence of LPS (*Salmonella typhimurium*, 1 mg/l, Sigma-Aldrich). After cultivation, the concentration of TNF-α in the supernatant was measured with ELISA (Invitrogen). The nuclear proteins were extracted from stimulated RAW264.7 cells by a nuclear extract kit (Active Motif, Rixensart, Belgium) and used to quantify the DNA binding activity of p65 subunit using the TransAM NF-κB family transcription factor assay kit (Active Motif). In NF-κB assay, only the concentration with the strongest immunomodulatory properties of Lc was used, i.e. 10 pg/l. All assays were performed according to the manufacturer's recommendation.

### Evaluation of microbiota changes by pyrosequencing

Stool samples from PBS or Lc-treated mice, on day 0, 28 (just before DSS administration) and 35 (the last day of experiment) were collected. Total DNA from these samples was then isolated with ZR Fecal DNA Kit™ (Zymo Research Corp., Orange, CA) according to the manufacturer's recommendation.

DNA was subsequently gel-purified and PCR was performed in triplicate for each primer pair, and pooled to minimize random PCR bias. The reaction mixture contained 1 µl of DNA (10 ng/µl), 1.5 mmol/l MgCl_2_, 0.2 mmol/l of dNTPs, 1× PCR buffer and 1 U platinum TAQ DNA polymerase (Invitrogen) and 0.40 µmol/l of forward modified primer consisting of 454 adaptor A (5′-CCATCTCATCCCTGCGTGTCTCCGACTCAG-3′; Genome Sequencer FLX system), unique 10-base tag sequence (ATATCGCGAG, CGTGTCTCTA, CTCGCGTGTC, TAGTATCAGC, TCTCTATGCG) and universal broad-range bacterial primer 5′-AYTGGGYDTAAAGNG and 0.40 µmol/l of reverse primer consisting of adaptor B (5′-CCATCTCATCCCTGCGTGTCTCCGACTCAG-3′) and universal primer TACNVGGGTATCTAATCC. PCR conditions were as follows: 1×: 95°C, 3 min; 35×: 94°C, 50 sec; 40°C, 30 sec; 72°C, 60 sec; 1×: 72°C, 5 min and final hold at 4°C. The length of PCR product was checked on the agarose gel electrophoresis. PCR product was subsequently purified using magnetic beads (AMPure beads, Beckman Coulter Genomics, Danvers, USA). Concentration was measured on Qubit fluorometer (Invitrogen, Carlsbad, CA, USA). Equimolar amounts of PCR product from each sample were used for unidirectional 454 FLX amplicon pyrosequencing using LIB-L emPCR kits following the manufacturer's protocols (Roche Diagnostics, Basel, Switzerland).

### Metagenomic data processing

Flowgrams were processed using amplicon analysis option in data processing software from Roche. The sequencing resulted in 161551 overall number of reads. Quality trimmed sequences obtained from the FLX sequencing run were processed using RDP pyrosequencing pipeline. Beforehand, data files were depleted of chimeras by Black Box chimera Checker [24] using default settings. Processing involved aligning of sequences with fast, secondary-structure aware Infernal aligner, subsequent clustering with max distance of 3% based on complete-linkage clustering method and classifying of incurred clusters by naïve Bayesian rDNA classifier [25]. Bootstrap cutoff was set to 50%, which was sufficient for accurate classification at the genus level. Generated designations of clustered sequences together with their relative abundances within the given samples were used for comparing bacterial diversity.

### Statistical analysis

One-way analysis of variance (ANOVA) with Dennett's multiple comparison test was used to compare multiple experimental groups with the control group. Differences between two groups were evaluated using an unpaired two-tailed Student's t-test and deviation of values from hypothetical mean were calculated by one sample t-test. The data is presented as the mean ± standard deviation (SD) unless stated otherwise and differences were considered statistically significant at P≤0.05. GraphPad Prism statistical software (version 5.0, GraphPad Software, Inc., La Jolla, CA,USA) was used for analyses.

## Results

### Oral administration of lysate *L. casei* attenuate the acute colitis in BALB/c mice but not in SCID mice

In our previous study we showed that oral treatment with *L. casei* DN-114 001 attenuates the severity of acute experimental colitis [21]. To test if its lysate have similar activity, we pretreated mice with four weekly oral doses of Lc and induced colitis by DSS in BALB/c and SCID mice. Oral (Table 1A) but not parenteral (data not shown) administration of Lc is effective in preventing the acute DSS colitis in BALB/c mice, improving clinical and morphological markers of colitis. In contrast, when colitis was induced in SCID mice (Table 1A) pretreatment with Lc failed to improve acute colitis in all tested parameters. Also no significant effects of Lc were found when the model of chronic colitis was used (data not shown).

**Table 1:** Lc improves the severity of DSS-induced colitis in BALB/c, but not in SCID mice.

Mouse strain	Experimental group	Disease activity index	Colon length (cm)	Histological grade
BALB/c	DSS/PBS	2.80±0.68	6.35±0.62	1.59±0.54
	DSS/Lc	1.67±1.09***	7.14±0.34***	1.20±0.51*
SCID	DSS/PBS	1.95±1.66	6.63±0.55	1.2±0,84
	DSS/Lc	1.99±1.54	7.17±1.21	1.26±0.85

Values are expressed as means ± SD (5 BALB/c mice per group) of one representative experiment out of three independent experiments. Unpaired Student's t-test in BALB/c mice was used to evaluate the significance of differences between experimental groups and the PBS-treated control group (*P<0.05, ***P<0.001).

### Lysate of *L. casei* prevents the increase in intestinal permeability and preserves ZO-1 expression in acute colitis

**Figure 1:**
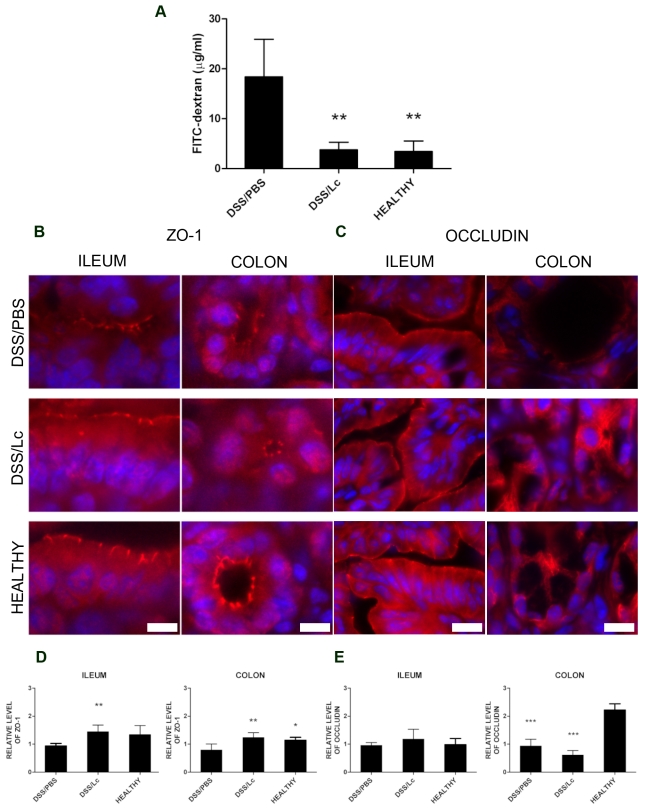
Oral treatment with Lc strengthens the gut barrier function as compared to PBS control mice. (A) Measurement of intestinal permeability by FITC-dextran. Serum levels of 4.4-kDa FITC-dextran 5 hour after administration by gavage in DSS/PBS, DSS/Lc-treated group and healthy controls. Immunohistological detection of tight junction proteins ZO-1 (B) and occludin (C) in representative sections of colon and terminal ileum from DSS/PBS-, DSS/Lc-treated group and healthy controls. Fluorescent signal of ZO-1 or occludin (red) is merged with DAPI counterstained nuclei (blue). mRNA expression of ZO-1(D) and occludin (E) evaluated in DSS/PBS, DSS/Lc treated group and healthy controls in colon and terminal ileum. RT-PCR was performed using TaqMan® gene expression assay for ZO-1. β-actin was used as the internal control. One-way ANOVA with Dunnett's multiple comparison test was used to evaluate the significance of differences between experimental groups and the DSS/PBS-treated control group (*P<0.05, **P<0.01, ***P<0.001). Data represent means (bar) ± SD (whisker) of five mice of one representative experiment out of three independent experiments. Scale bars are 10 µm in ZO-1 and 20 µm in occludin figures.

Increased intestinal permeability caused by impairment of the gut barrier function drives the pathogenesis of intestinal inflammation in both DSS-induced colitis and human IBD [26], [27]. To investigate the effect of Lc on the gut barrier function in acute DSS-induced colitis, we administered a single dose of FITC–dextran by gavage and measured the intensity of fluorescence in mouse serum 5 h later. Oral pretreatment with Lc significantly decreased the intestinal permeability to macromolecules on the last day of DSS (day 35) to the same extent as found in healthy mice (Figure 1A). One possible mechanism by which this effect could be mediated is the reinforcement of tight junctions. Previous studies have demonstrated that DSS causes the extensive decrease in ZO-1 expression and occludin redistribution and that this effect could be prevented by live bacteria or their components in the murine colonic epithelium [28], [29]. Therefore, we investigated whether treatment with Lc interferes with changes in the tight junction proteins production and distribution. As shown by immunohistochemistry and RT-PCR, treatment with Lc could completely prevent the loss of expression and changes in distribution of ZO-1 in both colon and terminal ileum (Figure 1B and D). Interestingly, in PBS-treated mice with subsequent induction of colitis (DSS/PBS) or in Lc-treated mice with subsequent induction of colitis (DSS/Lc) was a substantial loss of occludin in colon but not in terminal ileum (Figure 1C and E). Nevertheless, its distribution in colon seems to be slightly less affected in DSS/Lc- as compared with DSS/PBS-treated mice. Thus, we are able to demonstrate that the expression of ZO-1 in the colon and terminal ileum was significantly preserved following Lc treatment and probably contributes to reduced permeability of FITC-dextran. These findings suggest that treatment with Lc enhances the intestinal barrier function.

### Oral treatment with lysate of *L. casei* results in important changes in the gut microbial ecology

**Figure 2:**
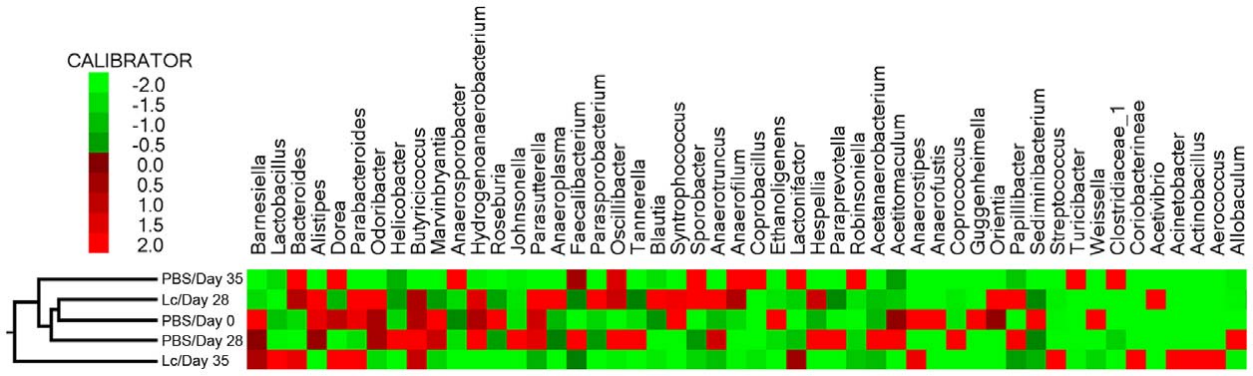
Oral treatment with Lc changes the intestinal microbiota composition. Normalized and z scored heat map and clustering dendrogram comparing relative abundance of the top 50 most abundant bacterial species in fecal microbiota of PBS (pool of 5 mice) and Lc-treated mice (pool of 5 mice) before the treatment (Day 0), before colitis induction (Day 28) and at the end of the experiment (Day 35). Horizontal columns represent the day of the experiment and or the treatment; vertical rows depict genus sorted from the most abundant species from left to right. The color scale for the heat maps is shown in upper left corner. The samples were clustered on the basis of their similarity by unsupervised clustering in the package CLUTO 2.1.1 (http://glaros.dtc.umn.edu/gkhome/cluto/cluto/download), as described previously [56].

Changes in the intestinal gut microbial ecology are expected to be associated with the state of disease and could be influenced by probiotic treatment [30]. To determine the impact of oral treatment with Lc on the intestinal microbiota, we used pyrosequencing of segments of genes for bacterial 16S rRNA. We collected feces before the treatment (day 0), before the colitis induction (day 28), and at the end of the experiment (day 35). We found that oral treatment with Lc resulted in significant changes in the intestinal microbial ecology (Figure 2). The frequently present genus in our fecal samples was a little-studied genus *Barnesiella*, from the Bacteroidetes phylum, one of the most abundant phylum in intestinal microbiota. The next most abundant genus with very well described capability to ameliorate intestinal inflammation *Lactobacillus* increased in abundance after exposure to DSS and the Lc treatment. This increase in abundance was not observed in the control PBS group. The *Bacteroides*, known to be increased during DSS-induced colitis, proliferated after intestinal inflammation, was induced in Lc and PBS treated groups. Moreover, there is an increase in the biggest group of genera from Clostridium cluster: butyrate producing *Butyricicoccus*, *Coprococcus* and *Anaerostipes*. Butyrate is crucial for energy homeostasis of mammalian colonocytes, capable to prevent their autophagy [31]. Dynamic changes in microbiota composition were observed before and during DSS administration in both Lc-treated and PBS-treated control group. Therefore, we can suggest that these microbial changes lead to improvement in gut barrier function and decrease susceptibility to intestinal inflammation by producing active substances such as lactate and butyrate.

### Oral administration of Lc changes the immune response of gut mucosa

**Figure 3:**
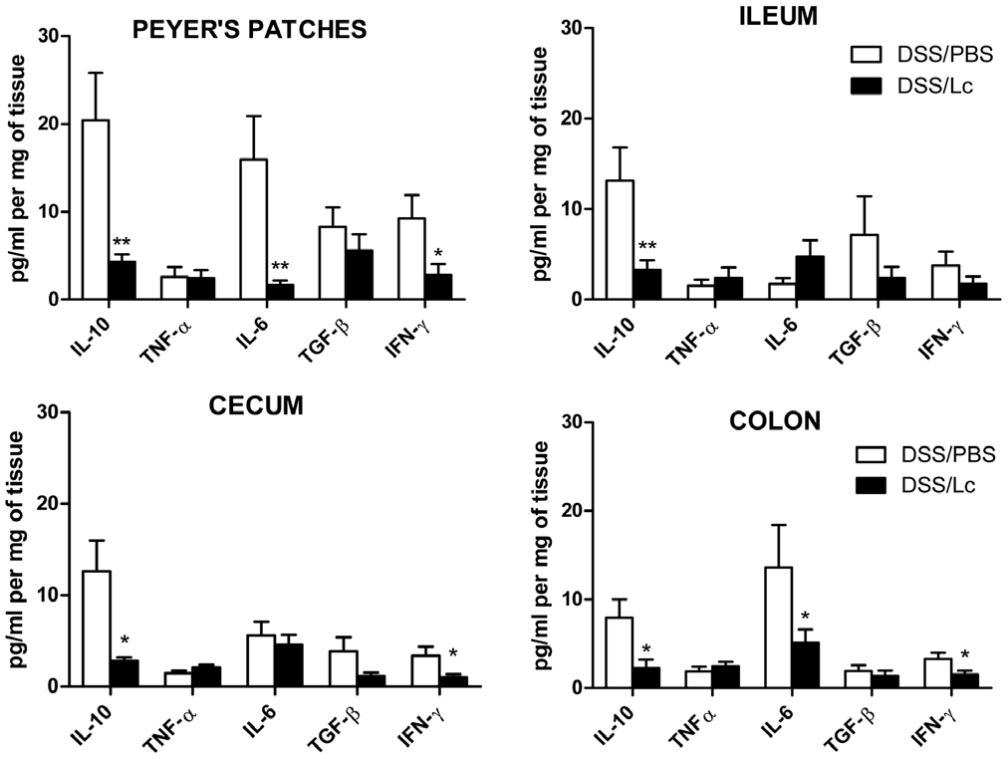
Pretreatment with Lc changes cytokine production in different parts of the gut. After DSS treatment and 24 hours cultivation, the production of cytokines TNF-α, TGF-β, IL-6, IL-10, IFN-γ differs in various parts of the gut as measured by ELISA. *P<0.05, **P<0.01 between DSS/PBS and DSS/Lc-treated mice in the same part of the gut was compared by unpaired Student's t-test (n = 10 per group).

Changes in cytokine microenvironment in the gut mucosa can influence the mucosal immune response to luminal antigens leading to the decrease of intestinal inflammation. Therefore, we investigated if the protective effect of Lc is associated with modifications in inflammatory response in the key compartments of the gut. We cultivated tissues from four distinct parts of the gut of either DSS/PBS or DSS/Lc-treated mice for 48 h and then measured the cytokines in supernatants by ELISA. We found that pretreatment with DSS/Lc decreased the production of pro-inflammatory cytokines (IL-6, IFN-γ) and anti-inflammatory cytokine IL-10 in PPs, cecum and colon as compared to DSS/PBS-treated mice (Figure 3). These results were confirmed at mRNA level by RT-PCR (data not shown).

### Lc treatment increased the number of regulatory T cells

**Figure 4:**
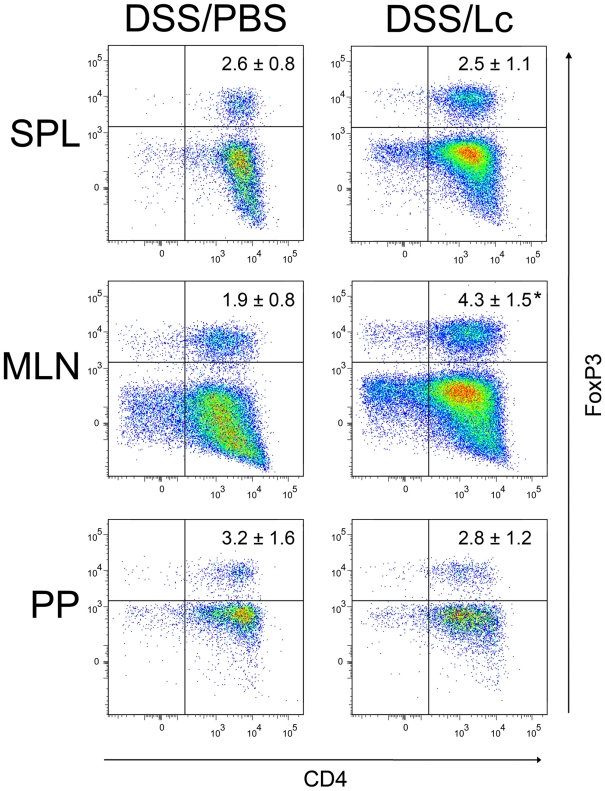
Oral treatment with Lc increases the number of CD4^+^FoxP3^+^ T_regs_ in MLNs. No significant changes were found in spleen or Peyer's patches. The plots shows the expression of CD4 *versus* FoxP3 on gated Th cells (CD3^+^CD8^−^), and the values within the plots represent the mean ± standard deviation of the total numbers of CD4^+^FoxP3^+^ T cells from one representative experiment out of three independent experiments (3–5 mice per group). One-way ANOVA with Dunnett's multiple comparison test was used to evaluate the significance of differences in numbers of CD3^+^CD8^−^CD4^+^FoxP3^+^ cells between DSS/Lc-treated groups and the DSS/PBS-treated (control) group (*P<0.05).

Since the intestinal inflammation in acute DSS-induced colitis is triggered by microbial antigens [32], the induction of oral tolerance to microbiota could be the one of the potential mechanisms of Lc protective effects. As the oral tolerance is maintained mainly at the periphery by T_regs_ , we analyzed the changes in CD4^+^FoxP3^+^ T_regs_ in the spleen, MLNs and PPs of DSS/PBS-, DSS/Lc-treated mice. We found a statistically significant increase in T_regs_ in MLN of DSS/Lc-treated mice as compared to DSS/PBS-treated mice. There were no statistically significant differences in the numbers of T_regs_ in spleen and PPs between these groups (Figure 4).

### Lysate of *L. casei*, but not *L. plantarum*, decreases the production of TNF-α and down-regulates NF-κB activity in LPS-activated macrophages

**Figure 5:**
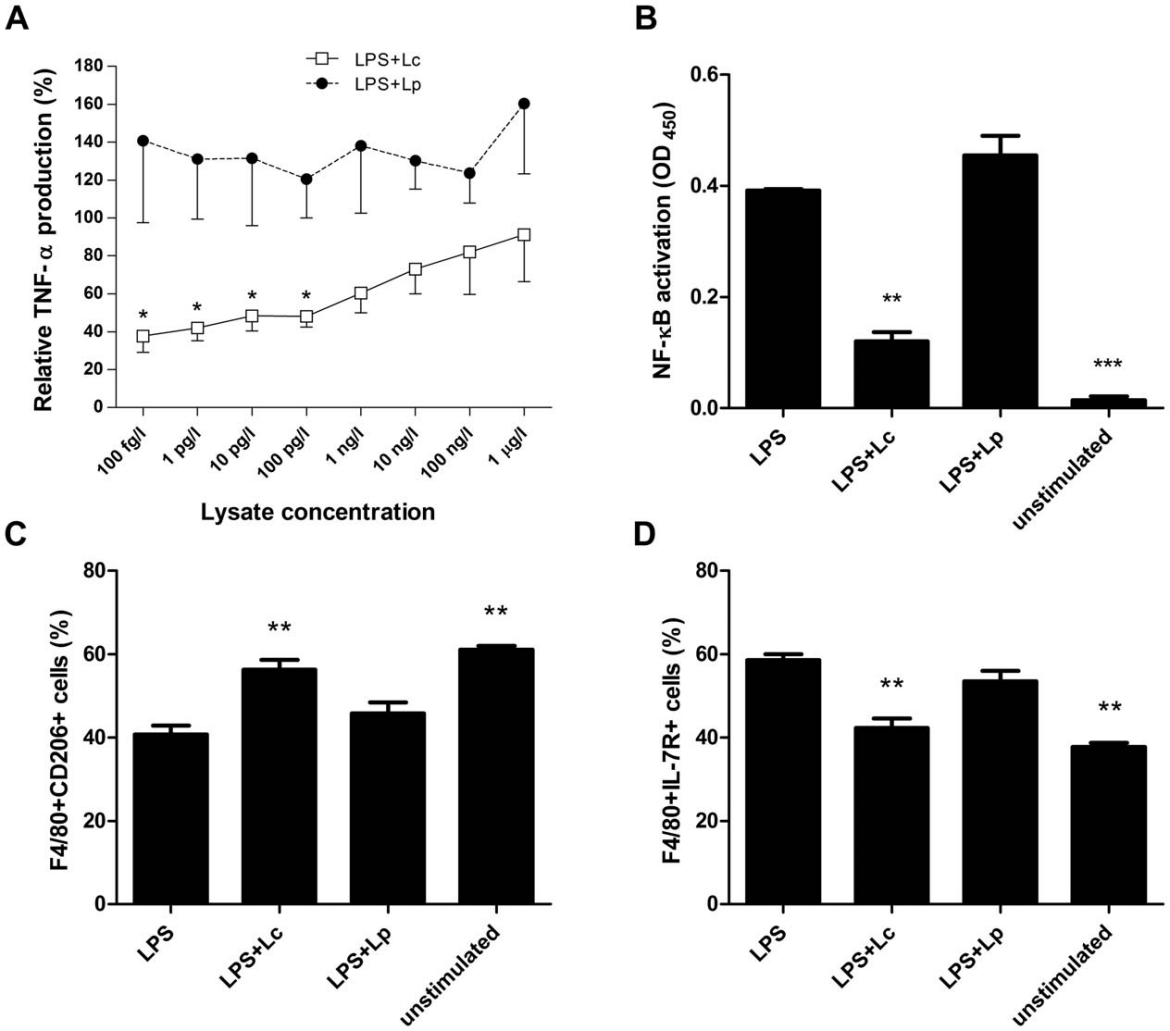
Lc exerts anti-inflammatory effect on LPS-activated macrophage cell line RAW 264.7. (A) Lc decreases the production of TNF-α in LPS-activated macrophages while Lp does not. TNF-α production by cells stimulated with 1 mg/l of LPS is set as 100% and data are expressed as means ± standard error of the mean of three independent experiments. *P<0.05: the means were compared against a hypothetical mean of 100% by one sample t-test (B) The effect of Lc on NF-κB binding activity in LPS-stimulated RAW 264.7 cells. Lc and Lp was co-cultured with LPS-activated cells for 24 h, and then the binding activity of NF-κB subunit p65 was analyzed by colorimetric assay. Data are expressed as mean ± standard deviation of three independent experiments. One-way ANOVA with Dunnett's multiple comparison test was used to evaluate the significance of differences between experimental groups and the LPS-treated cells group (**P<0.01, ***P<0.001). (C, D) Lc counteracts the LPS mediated M1 polarization. Expression of F4/80, CD206, IL-7R was determined by flow cytometry. One-way ANOVA with Dunnett's multiple comparison test was used to evaluate the significance of differences between experimental groups and the LPS-treated cells group (**P<0.01).

Because probiotics have an immunomodulatory effect on cells involved in innate immunity [33] and because the macrophages play a role in the pathogenesis of DSS-induced colitis [12], we analyzed the anti-inflammatory effect of Lc in LPS-activated macrophages *in vitro*. We found that doses below 100 pg/l significantly decrease the production of TNF-α by LPS-stimulated RAW 264.7 cells *in vitro*, while similarly prepared Lp did not (Figure 5A). Using the FACS analysis of cultured cells, we found that neither Lc nor Lp changes the viability of RAW 264.7 cells (data not shown). The treatment with either lysate of bacteria in the absence of LPS did not change the TNF-α production (data not shown), this data is in agreement with a study using *L. casei* 3260 [34]. As published by others [34], [35], this result suggests that Lc could interfere with the intracellular proinflammatory signaling cascade leading to activation of NF-κB transcription factor. To test this hypothesis, we isolated the nuclear extract from the untreated RAW 264.7 cells or from cells treated with either LPS (1 mg/l), or LPS with Lc and measured the activity of the NF-κB signaling pathway. Lc significantly decreased the NF-κB/DNA binding activity of p65 subunit as compared to the LPS-only or Lp+LPS treated cells (Figure 5B).

Since Lc treatment decreased production of TNF-α by LPS-activated macrophages, we decided to characterize macrophages further by investigating their stage of polarization by FACS. We found that M2 phenotype marker, the mannose receptor CD206 was significantly upregulated and M1 phenotype marker IL-7R downregulated in LPS+Lc treated macrophages as compared to either LPS or LPS+Lp treated macrophages. Therefore, Lc seems to counteract the LPS mediated M1 polarization. Neither Lc nor Lp without the addition of LPS changes the macrophage polarization.

## Discussion

Oral treatment with probiotic bacteria has emerged recently as a potentially useful therapeutic strategy for human IBD [5], [36]. However, the clinical utility of such approach remains controversial, as the link between specific mechanisms of action and therapeutic effects of specific bacterium has been difficult to establish. We have shown previously that repeated oral administration of probiotic bacteria *L. casei* DN-114 001 protects BALB/c mice from severe forms of acute intestinal inflammation [21]. In this study we demonstrated that not only live probiotic bacteria, but also its lysate protects BALB/c, but not SCID, mice from severe forms of DSS-induced inflammation.

The lack of protective effect in SCID mice suggests that mechanisms of adaptive immunity are essential for the beneficial effect of Lc. We did not find any changes neither in Lc-specific serum IgA, IgG and IgM, nor in gut SIgA during our experiments (data not shown), so we analyzed another mechanism executed by adaptive immune response, oral tolerance. Major role in this mechanism is played by T_regs_, whose protective role in inflammation control has been clearly established [37]. In this study, we found that Lc treatment leads to significant increase in T_regs_ in MLNs, but not PPs. This might be because MLNs are crossroads between mucosal and systemic immunity, because even naïve T cells (L-selectin expressing cells) can enter and after the interaction with gut-committed cells (α4β7-integrin expressing cells) from intestine became T_regs_ [38].

The intestinal barrier prevents viable enteric bacteria and the microbiota derived components from excessive interaction with the immune system. Here, we demonstrated that increase in intestinal permeability and the decrease in local ZO-1 expression, typical for DSS-treated mice, are both significantly improved by oral application of Lc. These results are in agreement with several studies showing that *L. casei* and other probiotics can strengthen the gut barrier function [28], [39]. Probiotic *E. coli* Nissle 1917 provided protection against DSS-mediated leakiness and was capable to produce specific up-regulation of ZO-1 expression in the intestinal epithelial barrier [29]. In addition, treatment with probiotic mixture VSL#3, where one of included bacterial strain is *L. casei*, prevents changes in expression and distribution of tight junction proteins ZO-1 and occludin [40]. It is well known that inadequate function of intestinal barrier could lead to inflammatory and neoplastic diseases [41], [42]. The disruption of the gut barrier has been identified as one of the crucial steps in IBD pathogenesis, causing excessive host-microbiota interaction during the initial phases of the IBD [26]. Protection of the gut barrier from disruption by induction of changes in expression and distribution of tight junction proteins and mucus was proposed as a key mechanism of probiotic function [29], [43].

Several studies showed that there is a marked difference in the gut microbiota composition in IBD patients (“dysbiosis”) as compared to healthy individuals. These changes in microbiota composition, or presence of certain microbial species with increased virulence, cause or perpetuate the intestinal inflammation in IBD [44]. Here, we report that oral treatment with Lc significantly changes the composition of gut microbiota. Similar effects have been already described as mechanisms involved in the probiotics-mediated protection from intestinal inflammation [29], [45]. Some of them are attributed to the fact, that probiotics can grow and colonize the gut, which could not be achieved with the non-living bacteria. The clear protective effect of bacterial lysate administration in intestinal inflammation is, therefore, rather indirect by shaping the gut microbial community or influencing the immune response. Nevertheless, similar mechanisms as in live bacteria could be involved to explain this effectiveness. Probiotics (or certain bacteria in general) can produce substances with antibiotic properties, such as bacteriocins, and molecules capable to signal to other members of the ecosystem to adjust their growth (quorum sensing modifiers), as recently reviewed [46]. These molecules could be present in the lysates of bacteria and, selectively modify the bacterial populations [47]. Moreover, certain probiotics can induce long-term production of anti-microbial peptides *in vivo*, which can shape the gut microbiota composition long time after the probiotic therapy has ended [48]. These mechanisms cause more favorable microbiota composition thus renders the Lc-treated mice less susceptible to intestinal inflammation.

By using the pyrosequencing technique we observed an increase in *Bacteroides* genus after induction of intestinal inflammation as shown previously [11]. DSS/Lc compared to DSS/PBS-treated group has shown a substantial increase in *Lactobacillus* genus which suggests that treatment with Lc promotes this genus among others. This effect could be caused by formation of niche ideal to lactobacilli. These and other differences in microbiota could be also explained by decreased inflammation in Lc-treated mice mediated by differing immunological mechanisms.

The cytokines produced in the gut mucosa greatly influence the resulting immunological outcome. The production of anti-inflammatory cytokines induces the mucosal unresponsiveness and tolerance and high levels of pro-inflammatory cytokines induce protective immune response and inflammation [49]. Here, we report that Lc treatment decrease the production of pro-inflammatory cytokines IL-6 and IFN-γ as well as anti-inflammatory cytokine IL-10 in both PP and the large intestine. This suggests that Lc can influence both the induction and effectors' functions of the mucosal immune system. We did not find significant decrease in local production of TNF-α, despite the clear differences in the colitis severity between Lc/DSS and PBS/DSS treated mice. This is consistent with our previous experiments, [11] and suggests that, despite being crucial pro-inflammatory cytokine produced by macrophages, TNF-α could be either exhausted or downregulated by IL-10, cytokine inhibiting TNF-α production, at this stage of colitis. Interestingly, since IFN-γ increases the gut permeability [50], a decrease in its local production can be responsible for strengthening of the gut barrier function as we found in Lc-treated mice. This is in agreement with findings that live *L. casei* can downregulate the pro-inflammatory mediators in the lamina propria of inflamed mucosa from Crohn's disease patients during *ex vivo* cultivation [51]. However, various strains of lactobacilli could differ in their immunological activities [52], and some lactobacilli are capable to induce T cells toward Th1 [53] or Th2 [54] immune responses.

Acute DSS colitis is believed to be driven initially by innate immunity mechanisms and, in particular, the role of macrophages has been proposed [12], [15], [32]. Therefore we tested the ability of bacterial lysates to decrease the inflammatory response of LPS-activated macrophages *in vitro*. We found that Lc, but not Lp, decreases the production of TNF-α, and the activation of NF-κB cascade and polarizes macrophages to M2 phenotype, suggesting a possible direct effect of Lc on the cells of the innate immunity. It is not excluded that negative regulators are involved in beneficial anti-inflammatory effects of probiotics [55].

In conclusion, our data provide evidence that even lysate of *L. casei* DN-114 001 can protect from induction of intestinal inflammation, thus confer a health benefit for the host. This is achieved by mechanisms that comprise: a) improvement in the gut barrier function, b) correction of the dysbiosis, and c) modulation of the mucosal immune response. These complex immunomodulatory properties of bacterial lysates may lead to the development of new therapeutic approaches for treatment of chronic intestinal inflammation. Moreover, oral administration of sterile bacteria, in contrast to live bacteria, may be safer in severely ill or immunocompromised patients.
